# P53 expression and micro-vessel density in relation with 5-year survival in patients with colorectal cancer

**DOI:** 10.1016/j.amsu.2020.08.006

**Published:** 2020-08-14

**Authors:** Ayad Ahmad Mohammed, Sardar Hassan Arif, Intisar Salim Pity

**Affiliations:** a Department of Surgery, College of Medicine, University of Duhok, Kurdistan Region, Iraq; b Department of Pathology, College of Medicine, University of Duhok, Kurdistan Region, Iraq

**Keywords:** Adenomatous polyp, Bleeding per rectum, Colonoscopy, Colorectal cancer, Microvessel density, P53

## Abstract

Colorectal cancers are among the commonest causes of cancer related morbidity and mortality worldwide. Adenomatous polyps which develop dysplastic changes are the main cause of invasive cancer.

P53 inactivation is a key genetic step in the occurrence of cancer. The degree of the formation of the blood vessels inside the tumor (microvessel density) increases the ability of the tumor to have local infiltration, metastatic potential and may reflect the tumor metabolic activity.

A total number of 104 patients who underwent surgeries for colorectal adenocarcinoma followed for 5 years. Patients were categorized into 4 categories based on tumor expression of P53 and micro-vessel density, 64.42% of patients were females, 58.7% died from colorectal cancer during the 5-year period, 37.5% had 5-years survival free from cancer, and 16.3% survived with recurrence, 28.8% had over expression of both P53 and MVD. A significant correlation was found between: P53 and MVD with the site of tumor occurrence being more significant with left-sided colonic tumors, the clinical stage being more significant with stage III disease, and with the survival rate being more significant in patients who died during this period, P values 0.025, 0.01, and 0.001 respectively.

Overexpression of P53 and MVD are associated with higher mortality and more advanced disease. We advise a more aggressive form of therapy for colorectal adenocarcinomas expressing high level of both factors and tumors with high expression of both factors may need modification in the chemotherapeutic drugs or radiation therapy with closer follow up than tumors having lower expression.

## Highlights

• Colorectal cancers are among the commonest causes of cancer related morbidity and mortality worldwide.
• P53 inactivation is a key genetic step in the occurrence of cancer.
• Microvessel density is the degree of the formation of the blood vessels inside the tumor.
• Microvessel density increases the tumor ability of local infiltration, metastatic potential and metabolic activity.

## 1 Introduction

Colorectal cancers are among the commonest causes of cancer related morbidity and mortality worldwide. It is still not well known what is the main cause of colorectal cancer but most authors accepted that they usually start as adenomatous polyps which develop dysplastic changes and if undiagnosed will change to an invasive cancer. The process of carcinogenesis involves the interplay of many other factors such as genetic factors with some environmental factors like diet [[1], [2], [3], [4], [5]].

One of the most important factors that determines the prognosis and the mortality is the tumor stage. When the tumor is confined to the wall of the bowel is staged I or II, stage III when the cancer spread to the regional lymph nodes, and stage IV when there is metastatic disease. Early stages have a better prognosis than late stages, operations that are done by more experienced surgeons may have also a better outcome [1,6].

Evidence present that mortality from colorectal cancer can be reduced through early detection and removal of adenomatous polyps which are precursors for cancer development, digital rectal examination and fecal occult blood test have not been shown to reduce the mortality rates.Genetic abnormalities may cause alteration in the tumor suppressor genes such as P53, adenomatous polyposis coli gene (APC) and some other genes. P53 inactivation is a key genetic step in the occurrence of cancer, in most tumors both alleles are suppressed [1,[7], [8], [9], [10], [11], [12]].

The degree of the formation of the blood vessels inside the tumor is called microvessel density, this increases the ability of the tumor to grow and to have local infiltration and have metastatic potential because it increases the chance of malignant cells to have an entrance to the circulation, and this may reflect the tumor metabolic activity. The P53 gene have been shown to regulate angiogenesis [[13], [14], [15], [16]].

The aim of this study is to determine the 5-year survival rates and the recurrence rates in relation to the P-53 expression and micro-vessel density in patients with colorectal cancer who underwent prior surgery.

### 1.1 Patients and methods

A total number of 104 patients who underwent various types of surgical procedures for colorectal adenocarcinoma for the last 5 years in single specialized surgical center were included in this study.

**Fig. 1:**
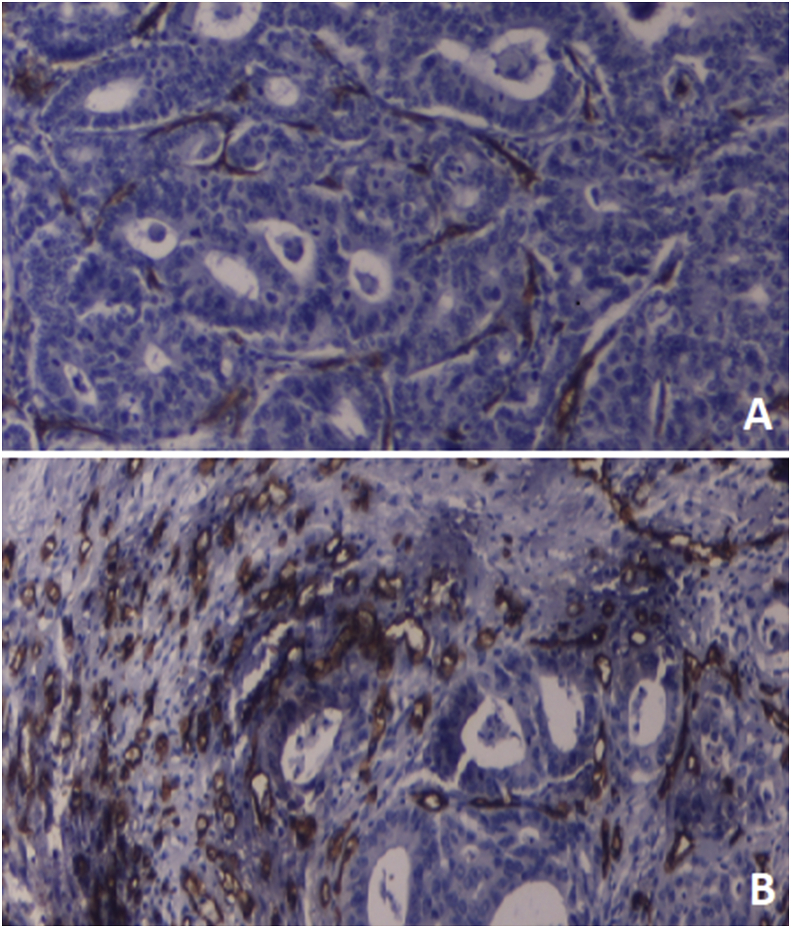
A microspic picture of colonic adenocarcinoma showing the microvessel density assessment, a) low MVD (<31), CD34 IHC, X200, b) High MVD (≥31), CD34 IHC, X200.

Assessment of microvessel density (MVD) came into clinical practice after the first experimental study which was reported by Weidner and Colleagues in 1991; they scanned the tissue sections at a low power (magnification, × 40). Blood vessels with a well-defined lumen (but not single cells) were taken into consideration for microvessels, and the average of 10 representative high power fields (magnification, × 400) was obtained. After that a double-scale (low and high) grading system was adopted using a single cut-off point (the median microvessel count was 31). Tumors with a count of less than 31 were considered as low MVD while those with equal or higher values than 31 were considered as high MVD, Fig. 1 [17].

Patients were categorized into 4 categories according to the presence or absence of P53 expression and micro-vessel density in the tumor. Category A included patient who had elevated expression of both P53 and micro-vessel density, category B included patients who had increase expression of P53 and decrease expression of micro-vessel density, category C included patients who had decreased expression of P53 and increased expression of micro-vessel density, and category D included patients who have decreased expression of both of them.

### 1.2 Study design and sampling

Patients with colorectal adenocarcinoma were consecutively assessed and prepared for surgery, 104 patients with different clinical stages of colorectal cancer were included in the study. The subjects were recruited from a single specialized surgical center in Duhok-Iraq, the operations were done by two surgeons who are experienced in the field of colorectal surgeries.

Patients with colorectal adenocarcinoma of all ages and from both sexes regardless of the type of the surgical procedure were included in this study, patients who presented with emergency presentations like intestinal obstruction or perforated large bowel due to tumors also were included in this study. Patients who refused to be included in the study and patients who skipped the 5 years follow up program were excluded.

All surgeries were conducted under general anesthesia. Patients who underwent scheduled operations had bowel preparation before the surgery, while emergency operations done without preparation due to the urgency of the surgery. Tumors were localized with colonoscopy before surgery in patients with scheduled surgery. The surgical procedure was either right hemicolectomy or extended right hemicolectomy in 26 (25%) patients, and left hemicolectomy, extended left hemicolectomy, and anterior resection of the rectum in the remaining 78 (75%) patients.

### 1.3 Diagnostic and measurement criteria

After surgery the samples were examined to determine the histopathological characteristics of the tumor and immunohistochemical analysis was done. Samples were categorized according to the presence or absence of P53 and micro-vessel density into 4 categories.

### 1.4 Statistical analysis

The descriptive purposes of the study were displayed in frequency and percentage for categorical variables, and mean and standard deviation for continuous variables. Patient survival and recurrence rates were determined and were correlated to the P53 and the micro-vessel density. The statistical calculations were done in Statistical Package for Social Sciences (SPSS 25:00 IBM: USA), p-value of less than 0.05 were considered significant.

Ethical committee registration: Ethical committee approval granted from the Duhok Directorate of General Health, Scientific Research Division at the July 30, 2019 with reference number 30072019–5, email: scientific.research@duhokhealth.org.

The research is registered according the World Medical Association's Declaration of Helsinki 2013 at the research registry at the 13th of December 2019, Research registry UIN: research registry 5794.

The work of this article has been reported in line with the STROCSS criteria [18].

## 2 Results

The majority of the patients that are involved in our study were females and most of the tumors were left side tumors; i.e. the descending, the sigmoid colon and the rectum. More than half of the patients (58.7%) died from cancer related causes during the 5 years follow up period and 16.3% had recurrence but survived more than 5 years. Table 1.

**Table 1:** Patients characteristics and the survival rates after 5-year follow up.

Patients characteristics; n = 104.	Frequency	Percentage
Gender; F (%) **Females** **Male**	67 37	64.42 35.57
Site of colon involvement; F (%) **Right side** **Left side + rectum**	26 78	25 75
Survival & recurrence; F (%) **Survival with no recurrence** **Survival with recurrence** **Deaths**	39 17 61	37.5 16.3 58.7

The most common category of our patients according to the P53 and the microvessel density was category A; i.e. both the P53 expression and the intratumoral microvessel density were elevated. Table 2.

**Table 2:** Patient categories based on the P53 and micro-vessel density expression.

Patient categories; n = 104	Frequency	Percentage
Category A (p53+, ↑MVD)	30	28.8
Category B (p53+, ↓MVD)	18	17.3
Category C (p53-, ↑MVD)	34	32.7
Category D (p53-, ↓MVD)	22	21.2
Abbreviations: MVD: **micro-vessel density.**		

Analyses showed a significant correlation to the site of involvement being more significant in left sided cancer (P value 0.025), while other factors such as the age and the sex showed no significant correlation. Table 3.

**Table 3:** Relation of the categories of the tumors to the age, sex and location of the tumor.

	Categories				P value
	A (30)	B (18)	C (34)	D (22)	
Age **< 50 years** **≥ 50 years**	13 (12.5%) 17 (16.3%)	9 (8.7%) 9 (8.7%)	11 (10.6%) 23 (22.1%)	7 (6.7%) 15 (14.4%)	
Gender: **Male** **Female**	7 (6.7%) 23 (22.1%)	3 (2.9%) 15 (14.4%)	15 (14.4%) 19 (18.3%)	12 (11.5%) 10 (9.6%)	
Location: **Left** **Right**	22 (21.2%) 8 (7.7%)	16 (15.4%) 2 (1.9%)	25 (24.0%) 9 (8.7%)	15 (14.4%) 7 (6.7%)	0.025*
*Only significant values displayed in the table.					

When correlating the categories with the clinical stage of the tumor, a significant correlation was detected between category A and stage III tumor (P value 0.01), while the other stages showed no such significance. Table 4.

**Table 4:** Relation of the different categories of the tumor to the stage of the tumor.

		Stage						p-value		
Category			I	II	III		IV			
A (30)			0	3	23*		4			0.01*
B (18)			1	5	8		4			
C (34)			9	16	5		4			
D (22)			2	5	8		7			
*Only significant values displayed in the table.										

The tumor related mortality was very significant in category A of patients (P value 0.001) suggesting that increased expression of both P53 and microvessel density are important 2 factors that determine the mortality of colorectal cancers. Table 5.

**Table 5:** Survival rate of the patients in relation to different categories of the tumor.

Category	Died	Survived	P value
A	23 (22.1%)	7 (6.7%)	0.001*
B	14 (13.5%)	4 (3.8%)	
C	18 (17.3%)	16 (15.4%)	
D	6 (5.8%)	16 (15.4%)	
*Only significant values displayed in the table.			

## 3 Discussion

Angiogenesis consists of the formation of new tumor related blood vessels, these vessels arise from the endothelium of the main blood vessels, when the size of the tumor reaches more than 2 mm it will induce new blood vessels formation which is required for its growth, this eventually will make the tumor to have a metastatic potential. This angiogenesis is the result of the interplay between many stimulatory and inhibitory factors [19].

The presence of genetic mutations will produce genomic instability that plays a key role in the process of carcinogenesis, genes that act as tumor suppressors are the most important in this process [20].

Microvessel density inside the tumor have been shown to be associated with an increased risk of tumor recurrence and increase metastatic potential of the tumor whether to the regional lymph nodes or to other organs particularly the liver. In some studies high level of intratumoral microvessel density had been shown to be a critical step in the adenoma transformation to carcinoma and has no clear important prognostic role [16,[21], [22], [23], [24], [25], [26]].

A key factor that regulates this intratumoral microvessel density is the vascular endothelial growth factor and some other genes, P53 mutation have also been found to be associated with high microvessel density because it increases the expression of factors that stimulate angiogenesis, decreases the factors that inhibit angiogenesis, and stimulates tumor cell proliferation [27,28].

In our study category A patients (elevated expression of both P53 and micro-vessel density) was significantly associated with shorter survival rates (P value 0.001) in comparison with other groups of patients where the levels of one of these values or both were low. This correlation also was found with the clinical stage of the disease (P value 0.01), patients in category A had more advanced stage at presentation when compared to other categories suggesting that P53 and MVD have a direct impact on the invasiveness of the tumor and the metastatic potential of the tumor. This correlation was proved by many authors and it is explained by the fact that increase angiogenesis will increase the ability of the tumor to spread locally, to the regional lymph nodes, and increase the distant metastatic potential of the tumor [19,29].

The size of the tumor has not been found to have a direct impact on the expression of the P53 and the intratumoral microvessel density, but some authors find that only microvessel density is associated with more advanced stage while the expression of P53 does not, larger size tumors are associated with a more advanced disease, in our study the relation was significant with the stage, most patients in category A had stage III disease at presentation [30,31].

The site of the tumor occurrence showed a significant correlation with P53 and MVD, left sided colorectal tumors showed a stronger association than right side tumors (P value 0.025). There is a difference between tumors that occur in the right and the left side of the large bowel at the molecular levels because the embryonic tissue of origin is different between both. Left side tumors comprise the most common type in our study (75%) and in most studies worldwide. Tumors in the left side of the colon and the rectum tend to have an earlier presentation than right sided tumors and show less aggressive behavior than right side colonic cancers, this is proved by many studies that included a large number of colorectal patients, although in our patients there was stronger expression of both P53 and MVD in left side colorectal tumors when compared to the right side tumors. As both factors are associated with more aggressive behavior of the tumor there are possibly many other factors that play a major role in the biological behavior and the aggressiveness of the tumors [32].

Other factors that increases the vascular endothelial growth factor are anoxia and estrogen hormone, most our patients that are involved were females; n = 67 (64.42%), although we didn't find any significant statistical relation between the microvessel density and the gender of the patient. Many studies have been done to find any relation with the estrogen hormone and the etiology and the oncologic behavior of colorectal cancer. In a study which was done to compare women with colorectal cancer who were taking estrogen with women not taking estrogen, the results showed that the estrogen has no role in the etiology of colorectal cancer but women who took the estrogen had more advanced disease when compared to the other group, the reason behind this is not clearly understood but this effect may be explained by the effect of estrogen of the bile acid concentration, estrogen mediated changes on the intestinal epithelium, and the changes of the levels of the insulin and insulin-like growth factor I [28,33].

Studies advocate that women aged more than 50 years who are taking the hormone replacement therapy should have more frequent bowel screening than the other groups, although in our patients we didn't find any correlation with the age [33].

MVD summarizes all the effects that act directly and indirectly on the angiogenesis and this factor probably have more important value in predicting the outcome than other factors, but despite its importance as a prognostic indicator, the intratumoral MVD have no role in guiding a specific therapeutic trial or a specific drug that suppress angiogenesis [14].

## 4 Conclusions

We concluded that increased P53 and high intratumoral MVD are associated with higher mortality and more advanced stage of the tumor. According to this conclusion we advise a more aggressive form of therapy in patients having colorectal adenocarcinomas expressing high level of both factors and tumors with high expression of both factors need modification in the chemotherapeutic drugs or radiation therapy and more close follow up than other tumors having lower expression.

## Research Registration Unique Identifying Number (UIN)

Researchregistry 5794

## Author contributions

Dr Intisar Salim Pity and Dr Sardar Hassan Arif did the data collection. Study design, analysis, and writing is done by Dr Ayad Ahmad Mohammed, Dr Intisar Salim Pity and Dr Sardar Hassan Arif. Final approval of the manuscript is done by Dr Ayad Ahmad Mohammed.

## Guarantor

The Guarantor is the one or more people who accept full responsibility for the work and/or the conduct of the study, had access to the data, and controlled the decision to publish.

## Source of funding

No source of funding other than the authors.

## Provenance and peer review

Not commissioned, externally peer reviewed.

## Declaration of competing interest

There is no conflict of interest to be declared.

## Appendix A Supplementary data

Multimedai component.1 Multimedai component.1
